# Remembering Professor Peter A. Leggat, AM, ADC (1961–2023) †

**DOI:** 10.3390/tropicalmed9020028

**Published:** 2024-01-24

**Authors:** Colleen Lau, John Frean

**Affiliations:** 1 Australasian College of Tropical Medicine, School of Public Health, Faculty of Medicine, University of Queensland, Brisbane, QLD 4006, Australia; colleen.lau@uq.edu.au; 2 Parasitology Reference Lab, Centre for Emerging Zoonotic and Parasitic Diseases, National Institute for Communicable Diseases, University of the Witwatersrand, Johannesburg 2192, South Africa

Professor Peter Leggat, the Immediate Past President of the Australasian College of Tropical Medicine (ACTM), passed away peacefully in Brisbane on 20 September 2023.

Peter was a much respected and beloved friend and colleague in the world of travel medicine and tropical medicine and one of Australia’s greatest contributors to these fields. He was also a wonderfully supportive and inspirational mentor for many in Australia and around the world. Always a gentleman, Peter’s quiet wisdom and calm guidance will be sorely missed by so many of us. Peter was a medical doctor, scholar, teacher, enabler, innovator, and most of all, a great leader. He had many talents, skills, areas of expertise, and countless accolades.

As a Founding Fellow of the ACTM in 1991, Peter contributed enormously to the college over many decades. He served five terms as President of the ACTM (1996–1998, 2002–2004, 2006–2008, 2016–2018, and 2020–2022) and held executive positions as Honorary Secretary, Honorary Treasurer, and Dean of the ACTM Faculty of Travel Medicine. In 2016, he was instrumental in establishing the ACTM’s peer-reviewed journal, *Tropical Medicine and Infectious Disease*, and as Deputy Editor-in-Chief led the journal to achieve its first impact factor, an impressive 3.711. Peter served as a Guest Editor for two Special Issues on COVID-19 that were later published in book form (Peter A. Leggat, John Frean, and Lucille Blumberg, Eds. *COVID-19: Current Challenges and Future Perspectives*. 2022. MDPI, Basel, Switzerland; Peter A. Leggat, John Frean, and Lucille Blumberg, Eds. *COVID-19: Current Status and Future Prospects*. March 2023. MDPI, Basel, Switzerland) and was a Guest Editor for the Special Issue in which this tribute appears.

After over 30 years of service at James Cook University (JCU), including many leadership roles, Peter recently retired as Professor Emeritus and Director Emeritus of the World Health Organization Collaborating Centre for Vector-borne and Neglected Tropical Diseases at JCU. As an academic researcher, Peter published over 500 journal papers (with over 9000 citations), more than 100 book chapters, and 30 books and presented more than 400 papers at national and international conferences.

In addition to his leadership roles at the ACTM and JCU, he was the Immediate Past President of the International Society of Travel Medicine, a member of the Australasian Public Health Medicine Council, a member of the Expanded Board of the International Federation for Tropical Medicine, Dean of Education for the Australasian College of Aerospace Medicine, Director of the World Safety Organization Collaborating Centre for Aerospace and Travel Health Safety, Distinguished International Fellow of the American Society of Tropical Medicine and Hygiene, and a Fulbright Alumni Adviser to the Australian–American Fulbright Commission. Peter was also a Medical Officer in the Australian Army, a Colonel in the Australian Defence Force, and an Honorary Aide-de-Camp to the Governor-General of Australia. Despite so many impressive achievements in such diverse fields, Peter was always unassuming, humble, gracious, congenial, and ready to share his wisdom.

Peter’s exceptional service to the community was recognised by many highly prestigious awards. In 2013, Peter was very deservedly recognised as a Member of the General Division of the Order of Australia (AM) for his significant service to medicine as a specialist in the fields of tropical and travel medicine. Later, in 2021, Peter was honoured as a Knight of Grace of the Order of St John and presented with the corresponding award at Government House in Canberra.

